# Liquid Biopsy and Tissue Biopsy Comparison with Digital PCR and IHC/FISH for HER2 Amplification Detection in Breast Cancer Patients

**DOI:** 10.7150/jca.66567

**Published:** 2022-01-01

**Authors:** Suhong Xie, Yan Wang, Zhiyun Gong, Yuan Li, Wentao Yang, Guangyu Liu, Jianwei Li, Xin Hu, Yanchun Wang, Yin Tong, Peng Yuan, Yiran Si, Yikun Kang, Yong Mao, Xiaowei Qi, Yankui Liu, Jiajia Ou, Zhaoliang Li, Xin Pan, Zhaoqing Lv, Kavanaugh Kaji, Lin Guo, Renquan Lu

**Affiliations:** 1 Department of Clinical Laboratory, Fudan University Shanghai Cancer Center, Shanghai, P. R. China.; 2 Department of Oncology, Shanghai Medical School, Fudan University, Shanghai, P. R. China.; 3 Department of VIP Medical Services, National Cancer Center/National Clinical Research Center for Cancer/Cancer Hospital, Chinese Academy of Medical Sciences and Peking Union Medical College, Beijing, P. R. China.; 4 Department of Oncology, Affiliated Hospital of Jiangnan University, Wuxi, P. R. China.; 5 Questgenomics, No.12 Mozhou East Rd, Nanjing, Jiangsu, P. R. China.; 6 Gnomegen, 6440 Lusk Blvd, D207, San Diego, CA, 92121 United States.

**Keywords:** HER2, liquid biopsy, tissue biopsy, sensitivity, digital PCR

## Abstract

Two hundred twenty-four breast cancer patients with paired tissue and plasma samples were enrolled from 3 clinical centers to evaluate sensitivity and specificity of a digital PCR HER2 amplification assay. All patients were histologically confirmed diagnosis of locally advanced and recurrent or metastatic breast cancer with stage III/IV and had tissue HER2 status determinations using IHC/FISH. For the whole 224 advanced breast cancer patients, the sensitivity between dPCR in plasma and IHC/FISH in tissue samples is 43.75% (42/96), the specificity is 84.38% (108/128) and the overall concordance is 66.96% (150/224). Interestingly, when we looked at stage III, stage IV and recurrent or metastatic breast cancer separately, compared with IHC/FISH in tissue samples, the sensitivity of dPCR in plasma increases from 37.93% (11/29) for stage III to 41.67% (15/36) for stage IV cancer. Recurrent breast cancer patient had an increased sensitivity of 51.61% (16/31). This is consistent with our expectation sensitivity would increase concordantly as tumor burden goes up. On the other hand, specificity decreased from 92.68% (38/41) for stage III to 86.44% (51/59) for stage IV cancer. Recurrent breast cancer patient had a specificity of only 67.86% (19/28). This is, in part, due to inter- and intra-tumor heterogeneity. Many patients determined to be negative for HER2 amplification in tissue biopsy could have HER2 positive tumors at other sites, which was detected by the liquid biopsy. This study suggested the necessity of liquid biopsy for HER2 amplification detection and demonstrated digital PCR can be used as a companion diagnostic tool to determine HER2 amplification status. It also suggested that a liquid biopsy should follow a negative result from tissue biopsy to avoid false negative results especially for late-stage breast cancer patients and ones who experienced relapse or became resistant to current therapy. Future studies should focus on therapeutic effects on patients determined to be HER2 positive through liquid biopsy and collecting additional tissue biopsies to identify HER2 positive tumor when the original tissue biopsy and liquid biopsy don't agree.

## Introduction

Targeted therapy significantly improves the therapeutic outcome of patients who test positive in the corresponding companion diagnostic tests. For example, breast and gastric cancer patients who tested positive for human epidermal growth factor 2 (HER2) amplification would have improved survival on anti-HER2 therapy 1, 2. Non-small cell lung cancer patients would benefit from different generations of tyrosine kinase inhibitors, depending on the particular mutation identified from epidermal growth factor receptor (EGFR) mutation detection assays 3-6. Because patients with such mutations would not respond well to regular chemotherapy, it is critically important to avoid false negative test results, so those patients do not miss their opportunity to benefit from targeted therapy 7.

Currently, tissue biopsy is commonly used as a companion diagnostic tool to decide whether a cancer patient would benefit from targeted therapy. However, tissue biopsy results are influenced by inter- and intra-tumor heterogeneity, leading to false negative test results 8-10. The possibility of having false negative test results is higher for patients with advanced cancer or who have relapsed because they will have bigger and multiple tumors 11. For these patients, it is difficult to get multiple tissue biopsies to have conclusive results. Additionally, mutations not present in the original biopsy may arise as the cancer progresses 12. These temporal changes have no way of being detected because tissue biopsies are not repeated, according to clinical standards. Therefore, it is important to supplement traditional tissue biopsy with additional information.

One method that can fortify tissue biopsy results is liquid biopsy. Liquid biopsy detects a variety of cancer related mutations from circulating tumor DNA (ctDNA) 11, 13, 14. ctDNA contains DNA mutations, epigenetic changes, among other abnormalities which encompasses the genetic topography of tumors. This gives liquid biopsy the potential to circumvent the problems stemming from intra- and inter-tumor heterogeneity. HER2 is a companion diagnostic biomarker for anti-HER2 therapy. In 2016, Otsuhi et al. found that plasma HER2 correlates with tumor size in HER2 positive patients 15. For these patients, the HER2 copy number status was used to monitor the tumor's response to therapies 15. HER2 amplification might not have been detected via tissue biopsy for cases with large or multiple tumors, as that method of detection cannot identify all subpopulations of tumor. Additionally, as tumors evolve throughout treatment, HER2 positive tumors may be enriched with regular chemotherapy as they would not respond to the treatment. Acquired HER2 amplification could also happen during regular chemotherapy 12, 14. HER2 amplification detection through liquid biopsy allows healthcare providers to prescribe anti-HER2 therapy for patients with false negative test results for liquid biopsy. Additionally, the relative ease of collecting body fluid samples, when compared to the invasive nature of tissue biopsy, allows repeated tests to determine HER2 amplification status during cancer treatment. This is especially important for patients who originally tested negative for HER2 and for the monitoring of therapeutic effects on patients with positive test results treated with anti-HER2 therapy.

HER2 amplification is routinely determined by immunohistochemistry (IHC) or fluorescence *in situ* hybridization (FISH) on tissue biopsy 16, 17. Those are tests based on HER2 protein (IHC) or chromosome DNA (FISH) done on formalin-fixed paraffin-embedded (FFPE) tissue samples. While these tests are considered the gold standard, IHC has poor reproducibility and is influenced by laboratory errors due to poor standardization; FISH, while being more robust, is limited by spatial heterogeneity because it is performed at high magnification 18-20. To detect HER2 amplification status in ctDNA, digital PCR (dPCR) is a great candidate technology. dPCR has been designed and developed to improve on the sensitivity of qPCR. Traditional quantitative PCR is only capable of relative quantification 21. dPCR, on the other hand, achieves absolute quantification through its methodology of partitioning of samples fundamental difference in methodology. For digital PCR, a PCR reaction mixture is partitioned into 20K different micro-reactions, resulting in, theoretically, 0 or 1 molecules of target DNA or RNA in each micro-reaction if input amounts of target nucleic acids are proper 22. After thermocycling, if the target molecule is present, the micro-reaction will have positive fluorescence. Users can count the number of positive micro-reactions to determine how many copies there are in the sample tested. Thus, for HER2 amplification, copy numbers for HER2 and a reference gene will be determined independently through counting the number of micro-reactions. Possion distribution will be used to adjust for number of micro-reactions when more than one copy of HER2 or reference gene may exist. A ratio between HER2 and reference gene can then be calculated accurately through this absolute quantification process; this allows for the detection of small changes in amplification 23.

A previous study found that the coefficient of variance (CV) of plasma HER2 was between 2-3%, and the limit of detection was 2.36 copies per diploid gene 24. This is significant because if the HER2 copy number is greater than 2.36, dPCR can detect the copy number variation. This level of sensitivity is especially important for patients who develop HER2 mutations throughout the course of their cancer as the number of HER2 copies would be relatively low. Digital PCR also has an easy to set-up process, fast turnaround time, and easy data interpretation. This allows the technology to be used by more healthcare providers and would benefit a wider variety of patients.

The goal of our study is to evaluate if digital PCR can satisfy the needs of HER2 liquid biopsy through a clinical lens. The study design includes the following: 1. Compare the positive percent agreement (PPA) and negative percent agreement (NPA) the chip-based digital PCR assay in plasma samples with IHC/FISH results in paired tissue samples; 2. Confirm the cut off value we set for HER2 amplification based on analytical studies on contrived clinical samples; and 3. Evaluate and conclude if different cut off values/threshold need to be set for different stages of cancer due to differences with tumor burden. We expect the results to confirm that liquid biopsy can effectively reduce the number of false negative test results from tissue biopsy in late stage or recurrent breast cancer patients due to the influence of intra- and inter-tumor heterogeneity. This suggests the necessity of detecting HER2 amplification by liquid biopsy and that digital PCR can be used to determine the HER2 amplification status.

## Materials and methods

### Patient cohort

The study cohort included 224 patients enrolled from 3 medical centers. Advanced breast cancer was confirmed by common clinical methods. The plasma samples were collected from all patients, and the test results of paired tissue samples from the same patient at the same period were collected. The tissue sample included primary tumors and metastatic lesions. Tumor HER2 status detected by IHC and/or FISH and positive for HER2 defined as 2+ or 3+ by IHC or FISH positive. The studies were approved by the Institutional Review Board or Independent Ethics Committee associated with each study center. The general principles of the International Ethical Guidelines for Biomedical Research Involving Human Subjects, the International Conference on Harmonisation guidelines on Good Clinical Practice, and the Declaration of Helsinki were followed. All patients had signed informed consent.

### Plasma preparation and cfDNA extraction

10 mL peripheral blood was collected in a PAXgene Blood ccfDNA Tube (Cat No: 768115, Qiagen, Hilden, Germany). The collected whole blood was centrifuged as soon as possible at 1900 ×g for 15 minutes under room temperature. The supernatant was collected and centrifugated again at 1900 ×g for 10 minutes. The supernatant from the second centrifugation was the final plasma which was stored at -80 °C until use.

The cfDNA was extracted from the plasma using the Qiagen QIAamp Circulating Nucleic Acid Kit (Cat No: 55114, Qiagen) according to the manufacturer's instructions.

### HER2 amplification status analyzed using digital PCR

The HER2 amplification status was assessed by the ratio of plasma HER2 copy number to reference gene copy number (HER2 ratio), which was performed on a ProFlex 2X Flat PCR system (Cat No: 4484078, Thermo Fisher Scientific, Waltham, MA, USA) using the HER2 Amplification detection kit (Cat No: Q0137365402, Questgenomics, Nanjing, JS, China). A total 14.5μl dPCR reaction mixture was prepared with 5.8 μl cfDNA sample and RNase-free water (about 5ng cfDNA input), 7.25μl dPCR Master Mix and 1.45μl HER2 amplification detection reaction solution. The dPCR reaction mixture was loaded into chip wells using the Questgenomics Chip Loader and then was sealed and loaded onto ProFlex 2X Flat PCR system according to the manufacturer's instructions. The cycling condition was as follows: 96 °C for 10 minutes, 39 cycles of 60 °C for 2 minutes and 98 °C for 30 seconds, followed by a final extension step at 6 °C for 2 minutes. The chip images were captured with the Questgenomics Biochip Reader and further analyzed using the Cloud Software from Questgenomics. Negative controls with no DNA were included in each run. A HER2 ratio ≥1.3 was defined as positive HER2 amplification, and a HER2 ratio < 1.3 was defined as negative HER2 amplification.

### Statistical analysis

All statistical analyses were performed using SPSS software (version 19.0) and Graph pad prism 7.04 (GraphPad Software, Inc., La Jolla, CA, USA). A receiver operating characteristic (ROC) curve was used to evaluate the optimal cut-off point in consideration of Youden index and to balance the PPA and NPA and a low false negative rate was chosen as priority. Data are presented as means ± standard deviation. A P value less than 0.05 was considered to be statistically significant.

## Results

### Patients' Characteristics

**Figure 1:**
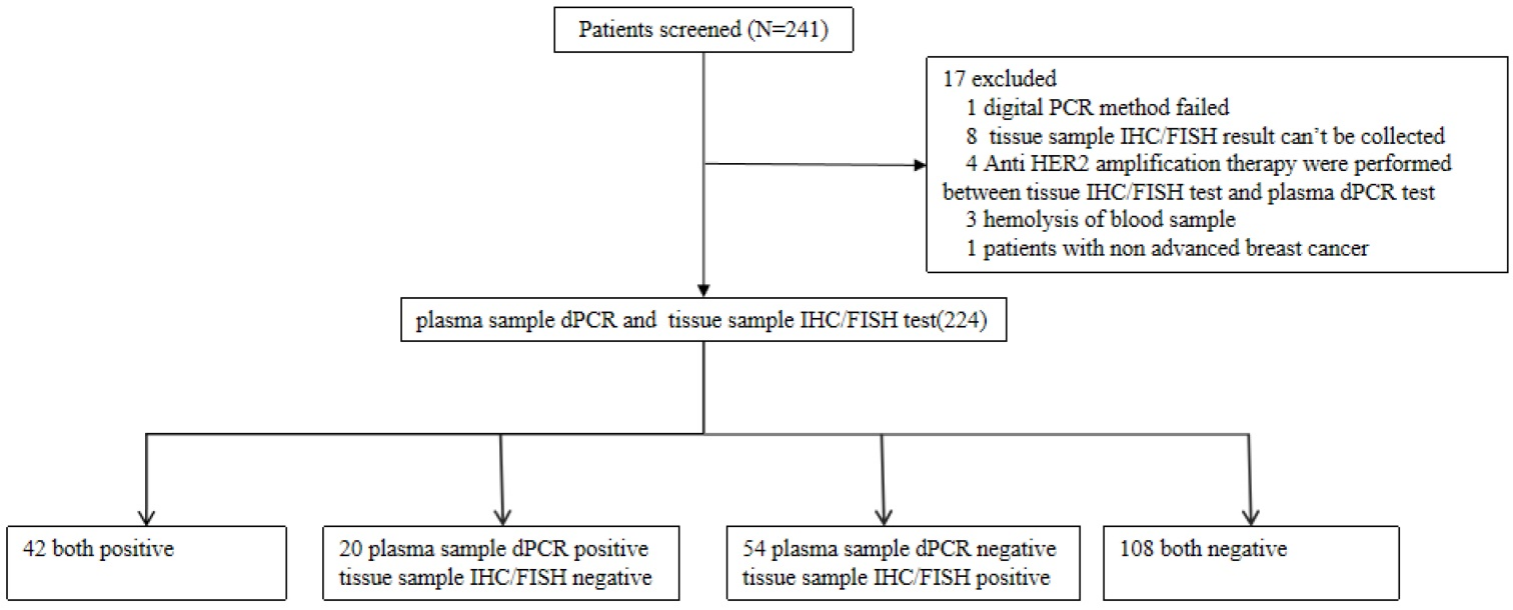
Flow chart of the analyses on enrolled patients.

The study included 241 patients from 3 clinical research centers that were screened for HER2 amplification. 17 patients that did not meet the selection criteria were excluded from the study and the samples of 224 remaining patients were analyzed. Of the 224 patients, the median age was 51 years old, 224 (100%) were females, 75 (32.89%) had stage III breast cancer and 149 (65.35%) had stage IV breast cancer. 210 out of 224 patients (92.11%) were diagnosed as invasive ductal carcinoma (Table 1). The flow chart of the analyses on these patients is shown in Figure 1. Stage III and IV patients are those who have received a diagnosis. Recurrent patients are those who have undergone surgery and have relapsed post-surgery.

We determined the plasma HER2 cut off and calculated the PPA, NPA, and overall compliance rate of HER2 status between digital PCR and IHC/FISH test. The receiver operating characteristic (ROC) curve had been drawn to set the cut-off value of plasma HER2 detection among different clinical stages. Youden index implicates the optimal PPA and NPA by analyzing the tissue HER2 status and plasma HER2 ratio.

### Comparison of HER2 amplification detection using digital PCR in blood ctDNA and IHC/FISH in paired tissue biopsy

According to the results of clinical validation studies on 98 clinical samples using ROC analysis, the cut-off value was determined to be 1.30 of HER2 over reference gene using the absolute quantification capability of digital PCR assay. At this cut-off value, the PPA, NPA, the positive predictive value (PPV), the negative predictive value (NPV) and the concordance were calculated based on the results of plasma detected by digital PCR assay and tissue samples by IHC/FISH. In this study, the prevalence was based off of the sample set and is not a reflection of a population.

For the 224 advanced breast cancer patients enrolled in this study, the PPA between dPCR in plasma and IHC/FISH in tissue samples was 43.75% (42/96), the specificity was 84.38% (108/128), the PPV was 67.74% (42/62), the NPV was 66.67% (108/162), and the concordance was 66.96% (150/224) (Table 2).

### Positive percent agreement, negative percent agreement and overall concordance are different for different stage cancer

We further analyzed the consistency of digital PCR in plasma samples and IHC/FISH in tissue samples in patients with stage III, IV and recurrent cancer respectively. We have found that for the 70 stage III breast cancer patients, the PPA between dPCR in plasma and IHC/FISH in tissue samples was 37.93% (11/29), the NPA was 92.68% (38/41), the PPV was 78.57% (11/14), the NPV was 67.86% (38/56) and the concordance was 70.00% (49/70) (Table 3).

For the 95 stage IV breast cancer patients, the PPA between dPCR in plasma and IHC/FISH in tissue samples was 41.67% (15/36), the NPA was 86.44% (51/59), the PPV was 65.22% (15/23), the NPV was 70.83% (51/72), and the concordance was 69.47% (66/95) (Table 4).

For the 59 patients with recurrent breast cancer, the PPA between dPCR in plasma and IHC/FISH in tissue samples was 51.61% (16/31), the NPA was 67.86% (19/28), the PPV was 64.00% (16/25), the NPV was 55.88% (19/34), and the concordance was 59.32% (35/59) (Table 5).

The PPA increases from 37.93% (11/29) for stage III to 41.67% (15/36) for stage IV cancer. And recurrent breast cancer patients had an increased PPA of 51.61% (16/31). The results showed that the PPA would increase concordantly as tumor burden increases. On the other hand, NPA decreased from 92.68% (38/41) for stage III to 86.44% (51/59) for stage IV cancer. Recurrent breast cancer patients had a NPA of only 67.86% (19/28). This suggests that the inter- and intra-tumor heterogeneity gives rise to this result, which also suggests the necessity of liquid biopsy for HER2 amplification detection.

### ROC analysis to determine cut-off value for different stage cancer

For the 70 dPCR in plasma samples of stage III breast cancer patients, the IHC/FISH in paired FFPE samples as reference, the cut-off value was recommended to be 1.28 with the maximum Youden index. The area under the ROC curve (AUC) was 0.6501 with the PPA and NPA of 37.93% (11/29) and 92.68% (38/41), respectively. The concordance between dPCR in plasma samples and IHC/FISH in tissue samples for stage III breast cancer was 70.00% (49/70) shown in Table 6.

When the cutoff was 1.30, the PPA and NPA of the detection results of stage III breast cancer patients were as same as that when cutoff was 1.28 (Table 3).

For the 95 dPCR in plasma samples of stage IV breast cancer patients, the IHC/FISH in paired FFPE samples as reference, the cut-off value was recommended at 1.58 with the maximum Youden index. The AUC was 0.7079 with the PPA and NPA of 36.11% (13/36) and 94.92% (56/59), respectively. The concordance between dPCR in plasma samples and IHC/FISH in tissue samples for stage IV breast cancer was 72.63% (69/95) shown in Table 7.

The PPA and NPA were 41.67% (15/36) and 86.44% (19/28) respectively when evaluated at the threshold at 1.30, The concordance between dPCR in plasma and IHC/FISH on FFPE tissue samples for advanced breast was 69.47% (35/59) shown in Table 4.

For the 165 dPCR in plasma samples of stage III and stage IV breast cancer patients, the IHC/FISH in paired FFPE samples as the reference, the cut-off value was recommended at 1.33 with the maximum Youden index. The AUC was 0.6839 with the PPA and NPA of 38.46% (25/65) and 90.00% (90/100), respectively. The concordance between dPCR in plasma samples and IHC/FISH in tissue samples for advanced breast cancer was 69.70% (115/165) shown in Table 8.

The PPA and NPA were 40.00% (26/65) and 89.00% (89/100) respectively when evaluated at the threshold at 1.30, The concordance between dPCR in plasma and IHC/FISH on FFPE tissue samples for advanced breast cancer was 69.70% (115/165) shown in Table 9.

For the 59 dPCR in plasma samples of recurrent breast cancer, the IHC/FISH in paired FFPE samples as reference, the cut-off value was recommended at 1.56 with the maximum Youden index. The AUC was 0.6285 with the PPA and NPA of 45.16% (14/31) and 89.29% (25/28) respectively. The concordance between dPCR in plasma samples and IHC/FISH in tissue samples for recurrent advanced breast cancer was 66.10% (39/59) shown in Table 10.

However, the PPA and NPA were 51.61% (16/31) and 67.86% (19/28) when evaluated at the threshold at 1.30, respectively. The concordance between dPCR in plasma and IHC/FISH on FFPE tissue samples for recurrent advanced breast cancer was 59.32% (35/59) shown in Table 5.

## Discussion

We have confirmed the cut off value for the digital PCR based assay to be at 1.30 for plasma samples. The PPA, NPA, and overall concordance of HER2 amplification status between digital PCR plasma samples and IHC/FISH paired tissue samples were calculated at this cut off value. An increase in PPA became evident as it increased from 37.93% (11/29) for stage III cancer patients to 41.67% (15/36) for stage IV and 51.61% (16/31) for patients who relapsed after surgery. This gradient is due to an increase in tumor burden, which would include an increase in number of tumors and the cumulated mass of multiple tumors combined. Therefore, the PPA is higher in late-stage cancer.

NPA, on the other hand, reflects an opposite trend of PPA, which started at 92.68% (38/41) for stage III breast cancer patients to 86.44% (51/59) for stage IV and 67.86% (19/28) for relapsed patients. This decrease in NPA is significantly higher than the increase in PPA for the same group of samples. This suggests that there are more factors contributing to it than an increase in tumor burden. This result also suggests that the decrease in NPA could not be due to false positive test results. On the other hand, this observed difference of NPA may be due to varying degrees of inter- and intra-tumor heterogeneity between the stages of cancer. Tissue biopsy normally takes one tissue sample from one tumor. If there are multiple tumors in the body, they would not be detected. Additionally, tissue samples do not reflect the mosaic of mutations present throughout a single tumor. It is very possible that if a small tissue sample is taken, HER2 amplification will not be detected even if amplification may exist elsewhere within that tumor due to spatial heterogeneity.

Besides the spatial heterogeneity, temporal heterogeneity was also reported by a plethora of studies 9, 15, 24, 25. After relapse or when patients develop resistance to the current chemotherapy, HER2 amplification may increase. That is when an additional HER2 liquid biopsy should be done. It is logical to assume that a HER2 liquid biopsy assay based on dPCR would identify HER2 positive patients even if they may have a negative result from tissue biopsy, and this is especially relevant for late-stage cancer patients. Therefore, liquid biopsy is necessary to help avoid many false negative results for HER2 amplification.

There have been discussions with oncologists during the process of the clinical study whether different cut off values should be arranged for different cancer stages. Having investigated data in detail, we have concluded that a single cut off value should be set at 1.30. As shown in the result session, although we have seen better PPA, NPA, and overall concordance if a different cut off value is selected based on the optimal Youden index, we believe it could be misleading as the discrepancy between test results from plasma and paired tissue samples may result from spatial and temporal tumor heterogeneity. This suggests that the reference method for tissue biopsy had a systematic error, and therefore may not be the optimal reference gold-standard for a ROC analysis, especially when it is expected to have a higher false negative rate due to increased tumor size and number of tumors for relapse cancer patients. Additionally, although increasing the cutoff value results in higher specificity, the degree to which the PPA is lowered makes increasing the cutoff value inadvisable. With the higher cutoff value, true positive datapoints are lost. This is especially harmful to recurrence and metastasis patients as they typically have higher degrees of inter- and intra-tumor heterogeneity and to patients with acquired HER2 positive status as they have lower copy numbers. The number of false negative results would be high and would misinform healthcare professionals leading to ill-informed treatment plans.

Liquid biopsy as a companion diagnostic tool is incredibly important for HER2 positive patients. Many patients who are negative for HER2 via tissue biopsy may be found positive with liquid biopsy; this is confirmed with a previous study which found that detection of the EGFR T790M mutation is comparable between liquid and tissue biopsy 26. This technology is especially important for patients who have relapsed, as a second tissue biopsy is typically not conducted. These patients would greatly benefit from continual monitoring. Liquid biopsy is the best method, out of current technologies, for this type of monitoring since the collection of samples is easier and less invasive than traditional tissue biopsy. For patients who are found to be negative with tissue but positive with liquid biopsy, another tissue biopsy should be taken. This is corroborated with National Comprehensive Cancer Network guidelines which state that if a patient is found negative via liquid biopsy, a tissue biopsy test should be ordered, if possible 27.

In conclusion, the newly developed digital PCR detection has high specificity and cost-effectiveness, which can be an effective tool to detect plasma HER2 status.

However, our study still has some limitations. Although it can be seen from the results that the tissue IHC/FISH results of recurrence or metastasis breast cancer patients should be false negative due to the inter or intra tumor heterogeneity, we have not been able to collect additional tissues to confirm that. Therefore, our future studies should focus on therapeutic effects on patients determined to be HER2 positive through liquid biopsy and collecting additional tissue biopsies to identify HER2 positive tumor when the original tissue and liquid biopsy don't agree.

## Figures and Tables

**Table 1:** The characteristics of enrolled patients

Index	3 clinical centers (N=224)	Ratio %
**Age**		
N	224 (0)	0%
Mean±Std	50.55±11.04	/
M(Q1,Q3)	51(43,58)	/
Min,Max	29,79	/
**Age**		
<50	105	46.88%
50-59	72	32.14%
60-69	38	16.96%
>69	9	4.02%
Total	224	100.00%
**Types of diseases**		
Invasive ductal carcinoma	210	92.11%
Invasive lobular carcinoma	4	1.75%
Invasive carcinoma, nonspecific	2	0.88%
Invasive/metastatic carcinoma	1	0.44%
Metastatic/invasive lobular carcinoma	1	0.44%
Medium to high grade ductal carcinoma *in situ*	1	0.44%
Metastatic breast cancer	1	0.44%
unknown	4	1.75%
Disease stage*		
Stage III	75	32.89%
Stage IV	149	65.35%
**Gender**		
Female	224	100.00%

*The number of patients listed are from the initial diagnosis. At the time patients participated in this study, 5 stage III and 54 stage IV patients relapsed.

**Table 2:** Concordance of dPCR in plasma and IHC/FISH on tissue HER2 detection in all enrolled breast cancer patients (N=224)

	HER2 with IHC/FISH in tissue		Total
	Positive	Negative	
ctDNA HER2 with dPCR in plasma			
Positive	42	20	62
Negative	54	108	162
Total	96	128	224

**Table 3:** Concordance of dPCR in plasma and IHC/FISH on tissue HER2 detection in stage III breast cancer patients (N=70)

	HER2 with IHC/FISH in tissue		Total
	Positive	Negative	
ctDNA HER2 with dPCR in plasma			
Positive	11	3	14
Negative	18	38	56
Total	29	41	70

**Table 4:** Concordance of dPCR in plasma and IHC/FISH on tissue HER2 detection in stage IV breast cancer patients (N=95)

	HER2 with IHC/FISH in tissue		Total
	Positive	Negative	
ctDNA HER2 with dPCR in plasma			
Positive	15	8	23
Negative	21	51	72
Total	36	59	95

**Table 5:** Concordance of dPCR in plasma and IHC/FISH on tissue HER2 detection in recurrent breast cancer patients (N=59)

	HER2 with IHC/FISH in tissue		Total
	Positive	Negative	
ctDNA HER2 with dPCR in plasma			
Positive	16	9	25
Negative	15	19	34
Total	31	28	59

**Table 6:** Concordance of dPCR in plasma and IHC/FISH on tissue HER2 detection in stage III breast cancer patients (N=70) with cut off value of 1.28

	HER2 with IHC/FISH in tissue		Total
	Positive	Negative	
ctDNA HER2 with dPCR in plasma			
Positive	11	3	14
Negative	18	38	56
Total	29	41	70

**Table 7:** Concordance of dPCR in plasma and IHC/FISH on tissue HER2 detection in stage IV breast cancer patients (N=95) with cut off value of 1.58

	HER2 with IHC/FISH in tissue		Total
	Positive	Negative	
ctDNA HER2 with dPCR in plasma			
Positive	13	3	16
Negative	23	56	79
Total	36	59	95

**Table 8:** Concordance of dPCR in plasma and IHC/FISH on tissue HER2 detection in stage III and IV breast cancer patients (N=165) with cut off value at 1.33

	HER2 with IHC/FISH in tissue		Total
	Positive	Negative	
ctDNA HER2 with dPCR in plasma			
Positive	25	10	35
Negative	40	90	130
Total	65	100	165

**Table 9:** Concordance of dPCR in plasma and IHC/FISH on tissue HER2 detection in stage III and IV breast cancer patients (N=165) with cut off value at 1.30

	HER2 with IHC/FISH in tissue		Total
	Positive	Negative	
ctDNA HER2 with dPCR in plasma			
Positive	26	11	37
Negative	39	89	128
Total	65	100	165

**Table 10:** Concordance of dPCR in plasma and IHC/FISH on tissue HER2 detection in recurrent breast cancer patients (N=59) with cut off value at 1.56

	HER2 with IHC/FISH in tissue		Total
	Positive	Negative	
ctDNA HER2 with dPCR in plasma			
Positive	14	3	17
Negative	17	25	42
Total	31	28	59

## Abbreviations

AUC: area under the (ROC) curve
ctDNA: circulating tumor DNA
CV: coefficient of variance
dPCR: digital polymerase chain reaction
EGFR: epidermal growth factor receptor
FFPE: formalin-fixed paraffin-embedded
FISH: fluorescence *in situ* hybridization
HER2: human epidermal growth factor 2
IHC: immunohistochemistry
NPA: negative percent agreement
NPV: negative predictive value
PCR: polymerase chain reaction
PPA: positive percent agreement
PPV: positive predictive value
ROC: receiver operating characteristic
